# Disseminated Basidiobolomycosis Caused by *Basidiobolus omanensis* in a Child with Acute Lymphoblastic Leukemia (ALL). Case Report and Literature Review

**DOI:** 10.1007/s11046-023-00820-3

**Published:** 2024-01-17

**Authors:** Laila Al Yazidi, Sharifa Al Sinani, Badriya Al Adawi, Marwa Al Riyami, Yasser Wali, Abdulhakeem Al Rawas, Buthaina Al Musalhi, Jacques F. Meis, Saif Al Housni, Ahmed Al-Harrasi, Abdullah M. S. Al Hatmi

**Affiliations:** 1 Sultan Qaboos University Hospital, Muscat, Oman; 2 Nizwa Hospital, Al Dakhiliya Region, Nizwa, Oman; 3 Department of Microbiology and Immunology, Sultan Qaboos University Hospital, Muscat, Oman; 4 Department of Pathology, Sultan Qaboos University Hospital, Muscat, Oman; 5 Department of Family Medicine, Sultan Qaboos University Hospital, Muscat, Oman; 6 Center of Expertise in Mycology, Radboud University Medical Center, Canisius Wilhelmina Hospital, Nijmegen, The Netherlands; 7 Natural and Medical Sciences Research Center, University of Nizwa, Nizwa, Oman

**Keywords:** Basidiobolomycosis, *Basidiobolus omanensis*, Disseminated, Children, Acute leukemia

## Abstract

Basidiobolomycosis is an uncommon fungal infection caused by the genus *Basidiobolus*. In immunocompetent children, it usually causes cutaneous infection and rarely affects the gastrointestinal tract, and it is extremely rare for the disease to spread. The present study reports the first case of disseminated basidiobolomycosis caused by *Basidiobolus omanensis* in a child with acute lymphoblastic leukemia who died as a result of uncontrolled infection and multi-organ failure despite surgical and antifungal therapy with L-AMB and voriconazole. A review of the literature yielded 76 cases, including the current case with the majority of which were reported as invasive gastrointestinal infection. The median age was 4 years (61 male and 15 female) and the majority of these children were from the Middle East (80%), specifically Saudi Arabia (45%). Most patients were treated with systemic antifungal agents (mostly itraconazole and amphotericin B). Surgical intervention was done in 25% of these patients and the death rate was 12%.

## Introduction

Human infections caused by *Basidiobolus* species are most common in immunocompetent people [1]. The *Basidiobolus* species' precise mode of transmission is currently unknown but the fungus has been found in reptile faeces [2]. The Entomophthorales order includes both *Basidiobolus* and *Conidiobolus* species, which are well known for causing limb lesions and subcutaneous and intrabdominal masses that result in obstructive gastrointestinal or renal symptoms [2]. *Basidiobolus* is a genus with eight phylogenetically distinct species, including *B. haptosporus*, *B. heterosporus*, *B. magnus*, *B. meristosporus*, *B. microspores*, *B. minor*, *B. omanensis*, and *B. ranarum* [3]. The majority of human infections are caused by *Basidiobolus ranarum* [4]. However, one case of *Basidiobolus omanensis* has been previously described from Oman causing gastrointestinal basidiobolomycosis (GIB [4]. Because of the disease's rarity, recognizing the microbiological and histopathological features can be difficult. Here we describe the second case of invasive basidiobolomycosis caused by *B. omanensis* in a child with acute lymphoblastic leukemia (ALL) and review the literature.

## Case Report

**Fig. 1:**
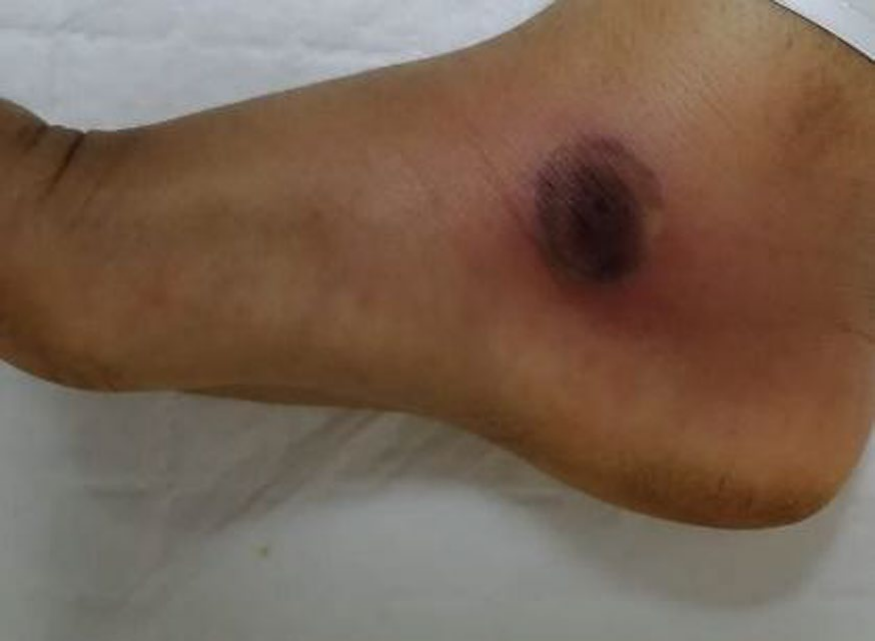
The necrotic skin lesion on the medial side of right foot

**Fig. 2:**
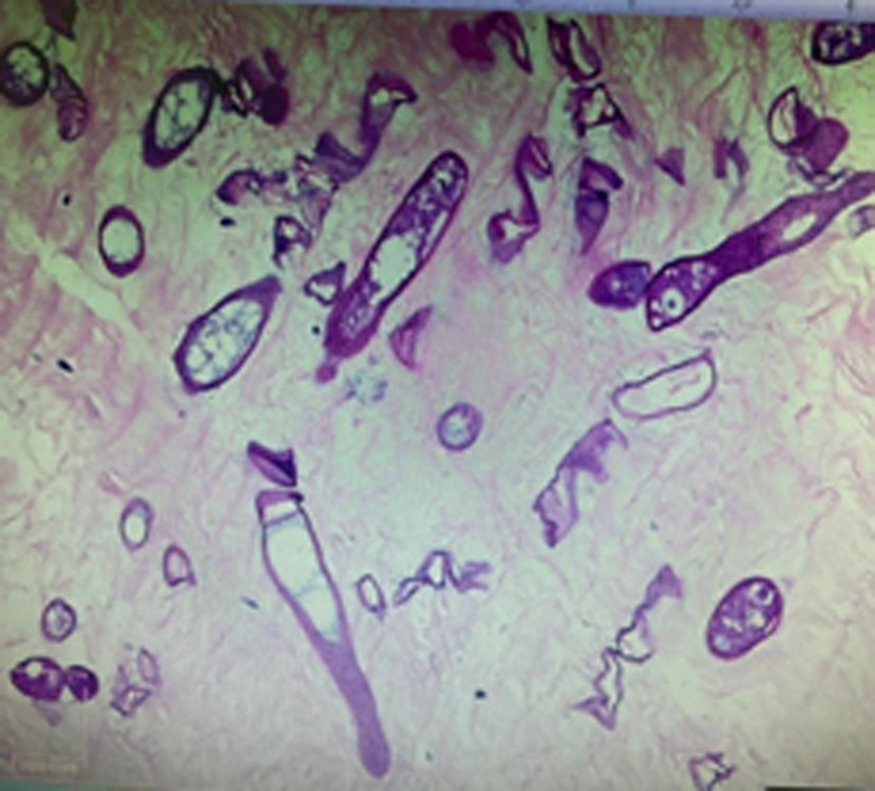
The histopathology of the skin biopsy from the right foot lesion which showed skeletal muscle and collagenous tissue with areas of necrosis but not much of inflammatory reaction with heavy growth of fungi of large, broad thin-walled non-branching hyphae with rare septa

**Fig. 3:**
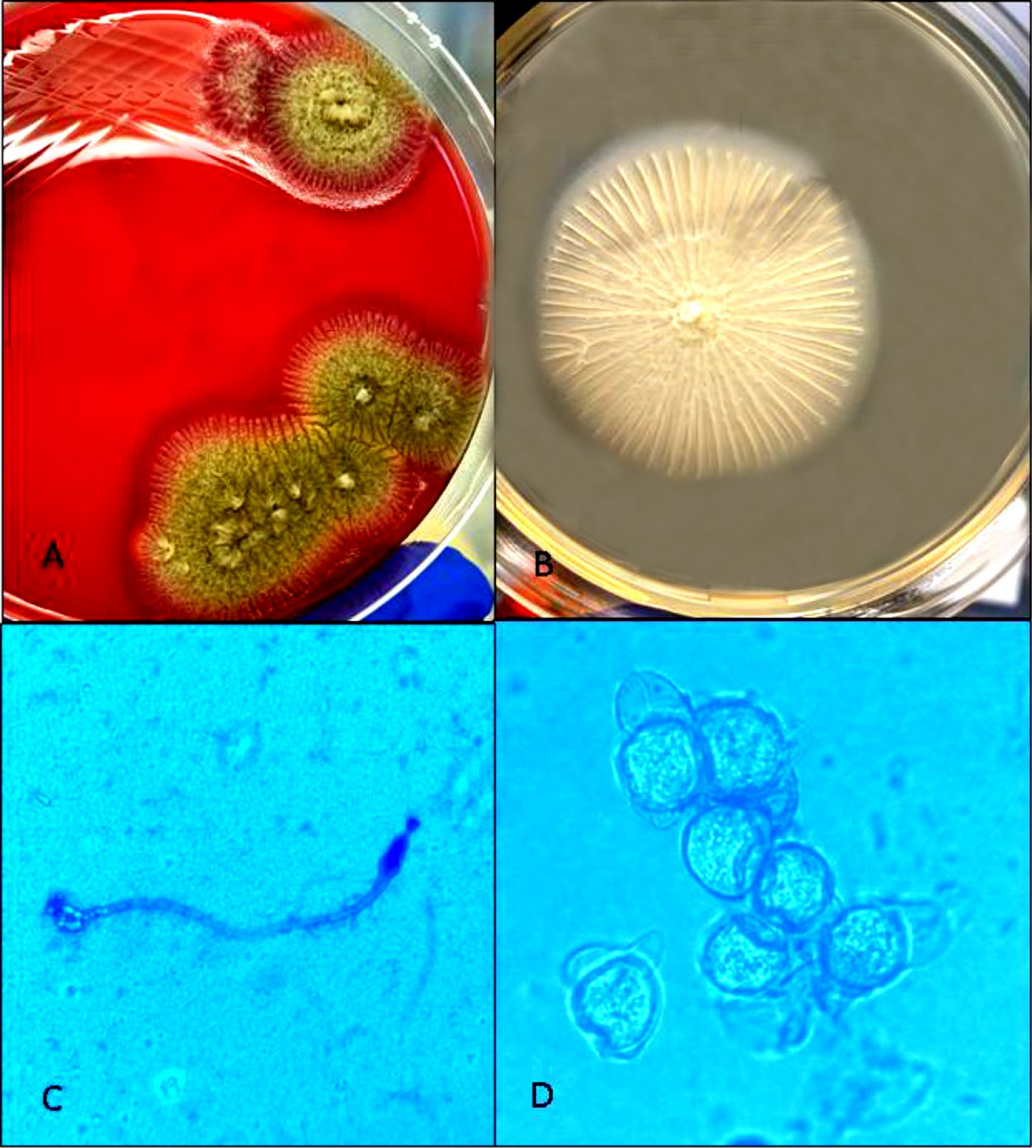
**A**. Primary culture on blood agar showing growth of mould. **B**. Colonial morphology of the mould upon subculture on Sabouraud agar. **C**–**D**. Microscopic features using lactophenol cotton blue stain demonstrating club-shaped spores with knob-like tips (**C**) and Zygospores with beak-like appendages (**D**)

A 3-year-old previously healthy boy was referred to Sultan Qaboos University Hospital (SQUH) in Muscat, Oman, for treatment of fever, bruises, and abdominal distention. At the time of presentation and examination, the patient had generalized lymphadenopathy, hepatosplenomegaly, pancytopenia, and circulating blasts in his peripheral blood smear. Shortly after admission, the patient was diagnosed with pre-B acute lymphoblastic leukemia (ALL) and began the UKALL 2011 regimen A treatment protocol. Since admission, the patient had been treated empirically with piperacillin-tazobactam (90 mg/kg/dose 6 hourly) for fever and neutropenia. One day after the start of chemotherapy, the patient developed a growing skin lesion on the medial side of his right foot, that progressed to a necrotic blackish lesion measuring 2 × 2 cm in size (Fig. 1). Meropenem and vancomycin were added to cover for the possibility of ecthyma gangrenosum due to *Pseudomonas aeruginosa* and methicillin resistant *Staphylococcus aureus*. Magnetic resonance imaging (MRI) was performed of his right ankle to determine the extent of the lesion. It revealed superficial cellulitis with necrotic areas as well as tenosynovitis without bone involvement. Because broad-spectrum antibiotics did not result in significant clinical improvement of the patient, the orthopedic team was consulted to discuss lesion debridement, and it was decided to perform a skin biopsy for histopathology and culture. The patient experienced severe abdominal pain and distension on the same day, followed by bloody diarrhea. His abdomen was noticeably distended and tender throughout. A contrast-enhanced computed tomography (CT) of the abdomen revealed a thickened, distended colon, suggestive of neutropenic enterocolitis and 3.5 × 2.7 × 2.9 cm large rectal collection, possible jejunojejunal intussusception. In addition, bilateral lower lobe lung nodular opacities suggestive of fungal infection were noticed. Liposomal amphotericin B (5 mg/kg daily) was added at this point to cover the possibility of disseminated fungal infection. The patient had an emergency laparoscopic laparotomy, showing a dilated, inflammatory transverse colon, enlarged lymph nodes, and a small amount of fluid in the pelvis but no tissue was obtained at this point in time. There was no intussusception, and there was no free air or feculent material in the abdomen. The skin biopsy from the right foot lesion showed multiple areas of necrosis as well as multiple fungal elements, including large, broad, thin-walled, non-branching hyphae with rare septa. There were no signs of inflammation or Splendore-Höeppli bodies (Fig. 2). These findings were highly suggestive of zygomycosis. After 3 days of incubation at 37 °C, the skin biopsy grew mould. The macroscopic and microscopic characteristics were typical for *Basidiobolus* species (Fig. 3). The skin lesion on the right foot was debrided and the majority of the underlying fascia was healthy and intact. At this stage, voriconazole was added to cover for basidiobolomycosis.

During the next two days, the patient's condition deteriorated, and his abdominal distention worsened, necessitating another laparoscopic laparotomy. Mild bilious ascites, a severely inflamed, unhealthy-looking transverse colon with multiple enlarged lymph nodes, proctitis with pale rectal wall, and white pale patches throughout the small bowel, sigmoid, and rectum, but no perforation, were observed intraoperatively. A right extended hemicolectomy, as well as an ileostomy and colostomy, were performed. The histopathology of the tissue sample from the transverse colon revealed multiple areas of ulcerated mucosa and complete thickness necrosis. Thin-walled, large broad non-branching hyphae with few septae and Splendore-Höeppli bodies were abundant in ischemic and necrotic areas. When using the Gomori methenamine silver (GMS) and periodic acid Schiff (PAS) stains, fungal hyphae were observed. The same fungal hyphae were found in the bowel wall, pericolic fat, and many blood vessels two weeks after admission and 7–10 days after starting chemotherapy and the patient being symptomatic, resulting in vascular thrombosis. Lymph nodes demonstrated reactive changes.

After starting granulocyte colony stimulating factor (G-CSF), chemotherapy was stopped, and co-trimoxazole 15 mg/kg/day was added as an adjunctive therapy. Despite supportive care, surgical debridement, and antifungal treatment, the patient died after approximately two weeks due to uncontrolled disease and multisystem failure.

**Fig. 4:**
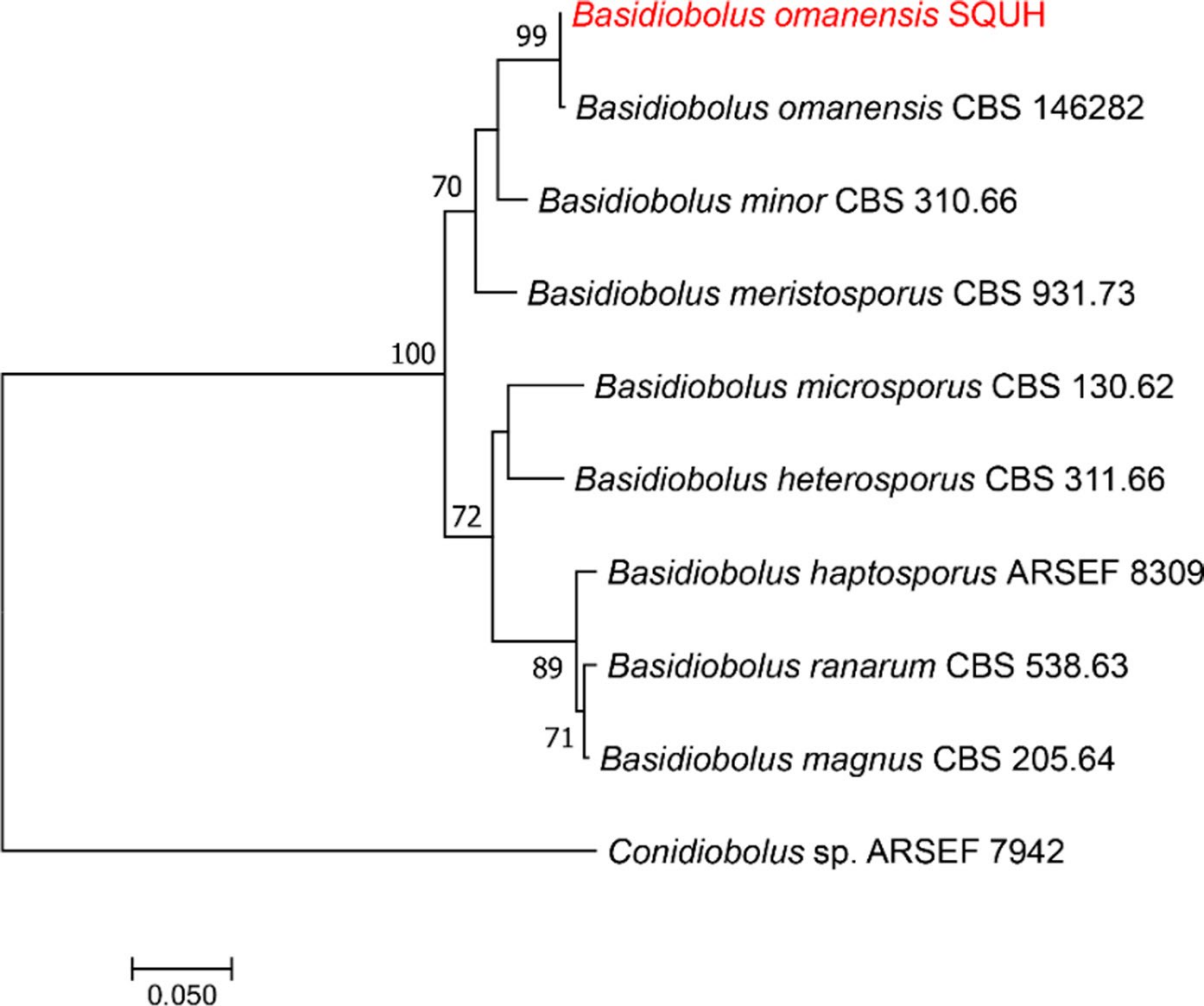
Phylogenetic tree generated by MLH analysis using ITS sequences of the *Basidiobolus* strains with closely related *Basidiobolus* species. Bootstrap support values above 70% are indicated at the nodes. Red colour indicates *B. omanensis* strain identified in this study (GenBank accession number xxxxxx). *Conidiobolus* spp. was used as outgroup

Further identification of the fungus was undertaken at the Centre of Expertise in Mycology of Radboud University Medical Centre/Canisius Wilhelmina Hospital, Nijmegen, The Netherlands. The isolate was subjected to direct DNA sequencing of the rDNA ITS region, which supported the identification as *B. omanensis* based on 99.64% similarity to the type of strain CBS 146281. For final identification, sequences of 10 reference *Basidiobolus* strains were included, representing eight *Basidiobolus* species, namely *B. haptosporus*, *B. heterosporus*, *B. magnus*, *B. meristosporus*, *B. microspores*, *B. minor*, *B. omanensis*, and *B. ranarum*. All sequences were aligned using MAFFT v. 7.127 (http://mafft.cbrc.jp). The best-fit model of evolution was determined by Model Test v. 0.1.1. Phylogenetic tree was constructed by using RAxML (v. 7.6.6). Maximum likelihood (ML) analysis was done with RAxML-VI-HPC v. 7.0.3 with nonparametric bootstrapping using 1000 replicates. The ITS tree was rooted with *Conidiobolus* sp. (ARSEF 7942) and edited in MEGA v. 7.1 (Fig. 4). The in vitro antifungal-drug susceptibility test, by the EUCAST. DEF.7.3.1 method, gave a minimum inhibitory concentration (MIC) for amphotericin B of 0.5 µg/mL, for isavuconazole 4 µg/mL, posaconazole and voriconazole, of 16 µg/mL.

## Literature Review

We collected data on children with basidiobolomycosis from 2005 to the present from PubMed, Elsevier, SpringerLink, WILEY, Web of Knowledge, and other databases using the following terms: "*Basidiobolus*" or "*Basidiobolus ranarum*" or "gastrointestinal basidiobolomycosis" and "children" collected. Cases with information on age, gender, country, site of infection, underlying disease, antifungal prophylaxis, treatment, and GIB outcome were included in the final analysis (Table 1).

**Table 1:** Shows a review of the literature on *Basidiobolus* species infections in pediatric patients published since 2005, including infections, clinical presentation, treatment, and outcome

Year	Country	Age (Years)/Sex	Site of infection	Surgery	Antifungal treatments	Outcome	Study
2005	India	8 F	Left thigh and leg	Biopsy	Itraconazole	Cured	Mathew et al. [15]
2006	Iran	1.5 M	Gastrointestinal	Resection	Amphotericin B then Itraconazole	cured	Fahimzad et al. [16]
2007	Saudi Arabia	13 M	Gastrointestinal	Hemicolectomy	Itraconazole	Cured	Hussein et al. [17]
2008	Iran	2.5 M	Gastrointestinal	Resection	Itraconazole	Cured	Geramizadeh et al. [18]
		2 M	Gastrointestinal	Resection	Itraconazole	Cured	
2008	India	11 F	Nasal cavity with ethmoid, sinuses extension	Nasal and sinus endoscopy with biopsy	Unknown	Lost to follow up	Singh et al. [19]
2010	India	3 M	Skin-thigh	Excision	Itraconazole	Cured	Anand et al. [20]
2011	Saudi Arabia	10 M	Gastrointestinal	laparotomy/resection	Itraconazole	Cured	El-Shabrawi et al. [8]
2011	Saudi Arabia	6 M	Gastrointestinal, intrahepatic	Biopsy only	Amphotericin B and Itraconazole	Cured	Rabie et al. [21]
		13 F	Gastrointestinal Recurrence of intrabdominal mass	Biopsy and cholecystojejunostomy	Amphotericin B and Itraconazole	Cured	
2011	Saudi Arabia	8 M	Maxillary and ethmoid sinuses	Biopsy	Amphotericin B then Itraconazole	Cured	Al Jarie et al. [22]
		10 M	Pulmonary	Bronchoscopy and Biopsy	Amphotericin B then Voriconazole	Cured	
2012	Iran	12 M	Gastrointestinal	Laparotomy	Amphotericin B then posaconazole	Cured	Arjmand et al. [23]
2012	Iran	15 months F	Gastrointestinal	Biopsy	Itraconazole	Cured	Geramizadeh et al. [24]
		5 M	Gastrointestinal	Biopsy	Itraconazole	Cured	
		5 M	Gastrointestinal	Biopsy	Itraconazole	Cured	
		2 M	Gastrointestinal	Biopsy	Itraconazole	Cured	
		16 months M	Gastrointestinal	Biopsy	Itraconazole	Died	
		13 months M	Gastrointestinal	Biopsy	Itraconazole	Cured	
		2.5 M	Gastrointestinal	Biopsy	Itraconazole	Cured	
		2 M	Gastrointestinal	Biopsy	Itraconazole	Cured	
2012	India	9 months F	Buttocks & perineum	Biopsy	itraconazole	Cured	Mendiratta et al. [25]
2012	Australia	5 M	Right chest wall skin lesion	Skin biopsy	Fluconazole/oral/unknown duration	Lost to follow up	Gordon et al. [26]
2012	Saudi Arabia	2 M	Terminal ileum, cecum and colon	Colonoscopy	Voriconazole	Cured	Saadah et al. [27]
2013	Saudi Arabia	12 F	Gastrointestinal	Hemicolectomy	Itraconazole	Cure	Al-Qahtani et al. [28]
		1.5 M	Liver abscesses	Percutaneous liver abscess drainage	Itraconazole	Died	
		9 F	Gastrointestinal	Biopsy	Itraconazole	Cure	
2013	Saudi Arabia	5 M	Gastrointestinal	Colonoscopy and biopsy	Voriconazole	Cured	AlSaleem et al. [29]
2013	Iran	12 M	Gastrointestinal	Rt hemicolectomy	Itraconazole and amphotericin	Died	Zahir et al. [30]
2013	Iraq	1.5 M	Gastrointestinal	Biopsy	Amphotericin	Died	Hassan et al. [31]
		1.5 M	Gastrointestinal	Rt hemicolectomy	Itraconazole	Improved	
2013	Saudi Arabia	4 M	Gastrointestinal	Laparotomy	Voriconazole	Cured	Al Asmi et al. [32]
2014	Iran	3 M	Gastrointestinal	Laparotomy and resection	Posaconazole and Itraconazole	Cured	Zabolinejad et al. [33]
2014	Saudi Arabia	11 M	Gastrointestinal	Biopsy	Voriconazole	Cured	Albaradi et al. [34]
2015	Iran	2 F	Multiple liver abscesses	Resection	Amphotericin	Cured	Geramizadeh et al. [11]
2015	Qatar	4 F	Gastrointestinal	Biopsy, resection	voriconazole	Cure	Mandhan et al. [35]
2017	Saudi Arabia	7 M	Gastrointestinal	Colonoscopy, Biopsy, colostomy	Voriconazole	Cure	Ageel et al. [36]
2017	Iran	5 M	Cecum, ileum ascending colon, liver	Colonoscopy, Biopsy, resection	amphotericin then posaconazole	Cure	Zekavat et al. [37]
2017	Togo	5 M	Skin over buttock, back and right flank	Biopsy	Ketoconazole	Cured	Saka et al. [38]
		3 M	Skin- right flank	Excision	Unknow	Cured	
2017	Saudi Arabia	16 months M	Terminal ileum ileocecal valve and small bowel	Laparotomy	Voriconazole	Cured	Al-Juaid et al. [39]
		19 Months M	Splenic flexure and descending colon	Biopsy	Itraconazole	Cured	
		22 Months M	Proximal ascending colon and cecum	Biopsy	Voriconazole	Cured	
		4 M	Terminal ileum and ileocecal valve	Laparotomy	Voriconazole + Surgical debridement	Died	
		8 M	Necrotizing granuloma	Biopsy	Voriconazole	Cured	
2017	Saudi Arabis	7 F	Gastrointestinal	Biopsy	Voriconazole	Survived	Almoosa et al. [40]
2018	Benin	3 M	Skin- lower limb	Biopsy	Unknown	Deceased	Brun et al. [41]
2018	India	7 M	perinephric abscess	Biopsy & perinephric fluid aspirate	Amphotericin B, oral itraconazole	Cured	Krishnamurthy et al. [42]
2018	Iran	2 M	Intestines, liver, ribs, lung, and the abdominal wall	Resection	Amphotericin B and itraconazole	Cured	Sanaei et al. [43]
2019	India	2 months M	Nephrostomy	Biopsy	Amphotericin B and itraconazole	Cured	Sharma et al. [44]
2019	India	4 M	Right distal arm	Biopsy	itraconazole	Lost to follow-up	Patro et al. 2019[45]
2020	UAE	22 months M	gastrointestinal	Endoscopy, Biopsy	Liposomal amphotericin B and voriconazole	Recovered	Kurteva et al. [46]
2020	India	3 M	Gastrointestinal	Biopsy	Voriconazole	Recovered	Ravindranath et al. [47]
2021	Iran	16 months M	Gastrointestinal	Laparoscopy, Biopsy	Amphotericin B	Cured	Mousavi et al. 2021[48]
2021	India	7 F	Dorsum of the left knee	Biopsy	Itraconazole	Cured	Sethy et al. [49]
2021	Oman	8 M	Gastrointestinal	Resection of ileocecal valve and right hemicolectomy	Voriconazole	cured	Al Harthy et al. [50]
2021	Oman	9 M	Gastrointestinal	Laparotomy, Biopsy	Unknown	Died	Al-Masqari et al. [51]
	Oman	5 F	Gastrointestinal	Right hemicolectomy	Amphotericin B then voriconazole	Cured	
	Oman	10 M	Gastrointestinal	Right hemicolectomy	Voriconazole	Cured	
2021	Saudi Arabia	8 M	Gastrointestinal	Biopsy		Cured	Al Haq et al. [10]
		4 M	Gastrointestinal	Multiple laparotomies, bowel resection	Voriconazole and itraconazole	Cured—short gut	
		2 M	Gastrointestinal	Laparotomy, Biopsy	Unknown	Cured	
		22 months M	Gastrointestinal	Laparotomy, Biopsy	Unknown	Cured	
		19 months M	Gastrointestinal	Laparotomy, Biopsy	Unknown	Cured	
		6 M	Gastrointestinal	Laparotomy, Biopsy	Unknown	Cured	
		5 F	Gastrointestinal	Laparotomy, Biopsy	Unknown	Cured—enterocutanous fistula	
		7 M	Gastrointestinal	Laparotomy, Biopsy	Voriconazole	cured	
		6 M	Gastrointestinal	Laparotomy, Biopsy	Unknown	cured	
		16 months M	Gastrointestinal	Laparotomy, Biopsy	Unknown	Cured	
		4 F	Gastrointestinal	Laparotomy, Biopsy	Unknown	Cured—Duodeno-colic fistula	
		3 M	Gastrointestinal	Multiple laparotomies	Voriconazole	Cured—Colocutaneousfistula and acquired atresia	
2022	Mexico	1 M	Transverse colon and terminal ileum	Laparotomy, Biopsy	Amphotericin B	Died	Fernandez and Vidales-Nieto [52]
2022	Saudi Arabia	29 months M	Gastrointestinal	Biopsy	Unknown	Lost to follow-up	Shaaban et al. [53]
2022	Saudi Arabia	4 M	Colonic basidiobolomycosis	Laparotomy, Biopsy	Voriconazole	Cured	Aljehani et al. [54]
2023	India	5 F	Cutaneous	Biopsy	Itraconazole	Cured	Rajkiran et al. [55]
2023	Oman	3 M	Gastrointestinal	Laparotomy, Biopsy	Amphotericin B	Died	Current case

## Discussion

Basidiobolomycosis is a rare fungal infection caused by the fungus *Basidiobolus ranarum*. *Basidiobolus meristosporus* [5], *Basidiobolus haptosporus* [6], and *Basidiobolus omanensis* are other *Basidiobolus* species that have been linked to gastrointestinal tract infections in humans [3]. The gastrointestinal tract is the first site of entry for *Basidiobolus* spores into the body in patients with GIB, and patients typically experience gastrointestinal symptoms. *Basidiobolus* is an insect pathogen that infects the entire insect body before being eaten by amphibians and reptiles. These fungi have the ability to infect humans through open skin, inhalation, or ingestion [7]. *Basidiobolus* can also cause chronic cutaneous and subcutaneous infections in the limbs, trunk, buttock, thigh, and perineum [1].

*Basidiobolus* spp. can spread to adjacent organs via transmural invasion in immunocompetent hosts [8], but in immunocompromised patients, *Basidiobolus* can easily spread via blood vessels to multiple organs distant from the site of infection [3]. *Basidiobolus* species have recently been reported, particularly *B. ranarum*, which affects children's GI tracts and are endemic in certain tropical and subtropical regions [9]. The most common route of infection in GIB is through ingestion of food contaminated by the fungus from soil or animal excreta. Ingestion of fungal-infested discharges from reptiles such as lizards (particularly the Gecko) and frogs is thought to be the route of infection in human gastrointestinal basidiobolomycosis (GIB) [10, 11].

After a postmortem examination of a 6-year-old boy with GIB affecting the ileum, transverse colon rectum, and urinary bladder, Edington reported the first pediatric case of GIB from Nigeria in 1964 [12]. Following that, two additional cases of GIB in children were reported from Brazil, with symptoms including abdominal pain, fever, and an epigastric mass, as well as involvement of the stomach, duodenum, transverse colon, pancreas, liver, and biliary system [13, 14]. Since then, several cases of GIB in children have been reported in the literature [8, 10, 11, 15–55]. We searched the existing English literature for all reported cases of basidiobolomycosis in children between 2005 and 2023, using the Medline database via PubMed, Embase via Scopus, ISI Web of Science, Science Direct, and Google Scholar. Despite the fact that pediatric gastrointestinal basidiobolomycosis is a rare infection, 76 cases of basidiobolomycosis have been reported in the English literature, mainly from Asia (69; 90%) [7, 9, 10, 15–55]. So far, the Middle East has been the source of the majority of reported cases with Saudi Arabia reporting the highest number of pediatric basidiobolomycosis (34; 45%) followed by Iran (19; 24%) and India (8; 11%). (Table 1) The median age of the affected children was 4 years of age. GIB is more common in adult males and children due to increased outdoor exposure [7, 49]. Around 80% of cases have been observed in people under the age of 20[7]. In this review, we found that 80% of the affected children were males and the youngest patient was two-month-old [51]. The majority of the reported children were healthy, and GIB was the most common form of basidiobolomycosis (61; 80%). Disease dissemination to other organs appeared to be uncommon (Table 1).

Basidiobolomycosis is usually chronic, indolent, and rarely spreads [56]. The patient had an unusual presentation. The patient was asymptomatic until a few days before admission, when the patient began to show signs of acute leukemia. The patient most likely had indolent asymptomatic GIB at first, but the immunosuppression caused by his underlying disease (ALL) and chemotherapy allowed the infection to quickly spread to the lungs and right foot. GIB clinical manifestations are extremely diverse, non-specific, and can mimic other conditions, resulting in delayed diagnosis and poor outcome [30]. GIB can mimic cancer, inflammatory bowel disease, parasitic infection, tuberous sclerosis, tuberculosis, and zygomycosis [10]. Pezzani et al. [57] examined 102 GIB cases reported in the medical literature and discovered that abdominal pain (86%) was the most commonly reported symptom, followed by fever (40%), and abdominal masses on physical examination (30%). In addition, eosinophilia was reported in 85% of these patients [14]. The most common symptoms of GIB in children are fever, abdominal pain, bloody diarrhea, and bowel masses [49].

The majority of laboratory diagnoses are based on typical histopathological changes and cultures, which are thought to be the most conclusive in establishing the diagnosis [58]. GIB diagnosis is typically difficult to diagnose due to the nonspecific clinical presentation, absence of predisposing factors, non-representative colonoscopic biopsies (involvement of non-mucosal layers of GI), non-specific inflammation or granulomatous reaction in histopathology, and histological features that resemble mucormycosis [58]. A biopsy of the infected tissue can reveal broad, unbranched hyphae with occasional septae (Fig. 2). Similar findings can be seen in patients with mucormycosis [59]. Histology of entomophthoramycosis caused by *Basidiobolus* and *Conidiobolus* species both exhibit the Splendore-Höeppli bodies [58]. It is distinguished by a widespread inflammatory response characterized by the presence of numerous eosinophils surrounding the hyphae. Despite the fact that molecular-based assays like PCR have higher sensitivity and specificity than microscopic and histopathological assays, as well as faster test results [60], these tests need to be standardized before they can be used in routine clinical settings.

Nonspecific radiological GIB findings such as diffuse wall thickening, and mass lesions can occur in a variety of conditions including inflammatory bowel disease and malignant tumors. [61]. In our patient, a CT scan showed a large rectal collection of jejuno-jejunal intussusception as well as bilateral lower lobe lung nodular opacities. Radiology and colonoscopy findings of thickening of the intestine or stomach walls frequently fail to confirm the diagnosis of *Basidiobolus* infection.

Potassium iodide (KI) and itraconazole are used to treat basidiobolomycosis. Amphotericin B, trimethoprim-sulfamethoxazole, and oral azoles such as ketoconazole (400 mg per day) were also used [44]. In some cases, resection of the affected bowel is required, followed by 6–12 months of systemic antifungal therapy [11]. However, approximately half of the reported *Basidiobolus* isolates from patients with basidiobolomycosis were amphotericin B resistant [62]. Our patient died with uncontrolled disease despite timely investigations, antifungal therapy, and surgical debridement for source control. Because of widespread disease in his gut, further surgical intervention was not possible. While waiting for susceptibility results, we used combination antifungal therapy with L-AMB and voriconazole. Mortality rate in children with basidiobolomycosis was 12% (Table 1).

To summarize, basidiobolomycosis is a rare fungal infection that mostly affects immunocompetent people, especially children. It is a very rare infection in immunocompromised people, and little is known about it. In this population, infection can worsen and spread. This case emphasizes the importance of thoroughly ruling out the presence of infections in immunocompromised patients.
